# Venous thromboembolism in COVID-19 patients

**DOI:** 10.1590/1677-5449.200107

**Published:** 2020-10-16

**Authors:** Fabio Henrique Rossi

**Affiliations:** 1 Instituto Dante Pazzanese de Cardiologia de São Paulo – IDPC-SP, São Paulo, SP, Brasil.

**Keywords:** COVID-19, SARS-CoV-2, thrombosis, anticoagulant, disseminated intravascular coagulation, prophylaxis

## Abstract

COVID-19 is a potentially serious respiratory disease caused by the SARS-CoV-2 virus that involves an increased risk of venous thromboembolism (VTE). Its pathophysiology is apparently related to an exacerbated inflammatory process and coagulopathy, verified by an increase in D-dimer, fibrinogen, and fibrin degradation products. Occurrence must be monitored, prevented, and treated according to existing recommendations and guidelines. The increased risk of thrombosis, and the association between this phenomenon and the most severe forms of the disease and death have prompted some groups to propose a more aggressive prophylactic and therapeutic approach. However, the risk-benefit profile of this type of conduct has not been defined and cases must be assessed individually, with a multidisciplinary approach. In this study, we review the main studies and evidence available to date on diagnosis, prophylaxis, and treatment of venous thromboembolism in COVID-19 patients.

## INTRODUCTION

In addition to alveolar lung injuries and acute respiratory failure, the COVID-19 pandemic, caused by the SARS-CoV-2 coronavirus, has also caused a high prevalence of cardiovascular diseases, in particular venous thromboembolism (VTE).^1^^-^3 The increased risk appears to be particularly associated with an exacerbated inflammatory reaction and exaggerated production of cytokines, in particular interleukin 6.^4^^,^^5^ Coagulopathy is detected in the form of high levels of fibrinogen, D-dimer (DD), and factor VIII, and prolongation of the prothrombin time (PT) and the activated partial thromboplastin time (aPTT), factors which are associated with poor clinical prognosis and death.^6^^-^8

These disorders predominantly occur in patients with risk factors, such as advanced age, obesity, systemic arterial hypertension, diabetes mellitus, heart diseases, lung diseases, cancer, thrombophilias, prior history of VTE, and other comorbidities, but they can also occur in children and younger people, which suggests that there is a genetic component involved. It should also be remembered that immobilization, dehydration, and the need for mechanical ventilation are factors that can contribute to the high prevalence. Prevalence rates during the different phases of the disease have not yet been fully defined; however, it has been observed that in the more severe forms, particularly in patients admitted to intensive care units (ICU), there is an elevated risk of thrombopulmonary embolism (TPE).^1^^-^3^,^^6^^-^8

The recommendations presented here are based on international consensuses on prophylaxis and treatment of VTE in patients without COVID-19,^9^^,^^10^ on editorials, on retrospective studies of cases series, and on expert recommendations and opinions on clinical practice with COVID-19 patients published recently on a fast-track basis, the majority without peer review.^11^^-^14 They should therefore be applied on a case-by-case basis by an experienced multidisciplinary team trained in use of these medications and procedures.

Anticoagulant therapy is associated with substantial benefits (reduction of risk of growth of the thrombus and fatal TPE during the acute disease and of recurrent VTE) and complications. Our objective is to provide an objective and practical presentation of recommendations based on current evidence on diagnosis, prophylaxis, and treatment of VTE in COVID-19 patients.

## DIAGNOSIS OF VTE IN COVID-19 PATIENTS

Patients with clinical suspicion of COVID-19, even when treated at home, and especially those who have fever, anorexia, and diarrhea, should be instructed about the importance of hydration, healthy nutrition, and exercising the limbs. If a clinical suspicion of VTE emerges (pain and swelling of lower limbs, chest pain, sudden exacerbation of dyspnea, etc.), the patient should be instructed to use telemedicine to consult an angiologist or vascular surgeon and, if necessary, attend a consultation in person. Nowadays, the majority of clinics are equipped with equipment for vascular Doppler ultrasonography (VDUS), which can help with diagnosis. In patients treated at home, there are no indications for requesting routine laboratory tests with the objective of identifying coagulopathy or elevated DD. For inpatients, the majority of authors recommend that these data should be verified periodically, although there are no studies that have investigated the cost-effectiveness of this practice.^3^^,^^4^ Diagnosis of VTE using imaging methods can be problematic in these patients while the pandemic is ongoing, because of exhaustion of hospital resources and the risk of contamination of people who are not Covid-positive, particularly ICU patients and the care team.^13^^,^^14^

For patients with elevated DD or a high degree of clinical suspicion of TPE and when there is no access to tomography, it has been recommended that bedside tests can be employed. Transthoracic echocardiogram can identify signs of right ventricle overload, which may suggest presence of TPE. Point-of-care VDUS can detect deep venous thrombosis present in the femoropopliteal axis.^13^^,^^14^

## PROPHYLAXIS IN PATIENTS NOT IN HOSPITAL

Patients who take antithrombotics should be instructed to continue taking them, but should be warned about the risk of drug interactions between the antithrombotics and medications prescribed for COVID-19, in particular antivirals and steroidal and non-steroidal anti-inflammatories. In general, pharmacological VTE prophylaxis is not indicated; however, prophylaxis with low molecular weight heparin (LMWH) can be considered, particularly among patients at increased risk of VTE (Caprini score > 8),^11^^-^14 as long as they are not at increased risk of bleeding. The preference for LMWH is because of its shorter half-life and fewer drug interactions than direct oral anticoagulants (DOACs). Vitamin K antagonists (VKA) should be avoided because of the difficulty of controlling international normalized ratios (INR), and DOACs should be administered instead whenever possible. However, patients with mechanical heart valves, valvular atrial fibrillation, or antiphospholipid antibody syndrome and also breastfeeding patients should be kept on VKA.^13^^,^^14^ The possibility of drug interactions between the different anticoagulants and continuous-use medications and those recommended for treatment of COVID-19 should be considered. Liverpool University maintains an extensive list of possible interactions between medications frequently used for COVID-19 on its website.

## PROPHYLAXIS AND TREATMENT FOR INPATIENTS

There is an elevated risk of VTE in all patients admitted with a confirmed diagnosis or clinical suspicion of COVID-19. All of them should be given pharmacological VTE prophylaxis unless there is an absolute contraindication.^9^^,^^10^^,^^13^^,^^14^ For patients who are at increased risk (Caprini score > 8), it is recommended that prophylaxis doses should be increased or doubled and, at hospital discharge, parenteral or oral pharmacological VTE prophylaxis should be maintained for a further 30 days.^13^^,^^14^ However, we should emphasize that this practice is not supported by the results of clinical studies and should be adopted on a case-by-case basis, considering the risks and benefits for each patient.

## RECOMMENDED DOSAGES^13^^,^^14^

The regular subcutaneous (SC) dose of LMWH is 30 mg 2x/day or 40 mg/day. For obese patients (body mass index [BMI] > 30 kg/m^2^), Caprini score > 8, and elevated DD (200-3500 ng/ml), consider 60 mg, SC, 2x/day; for patients with renal failure (creatinine clearance < 30 mL/min), consider 5000 UI of unfractionated heparin (UFH), SC, 3x/day; for patients with a history of heparin-induced thrombocytopenia, consider Fondaparinux, 2.5 to 5 mg, SC, 1x/day. For those with platelet counts < 30,000 or an absolute contraindication against anticoagulation, use intermittent pneumatic compression devices. For patients in a critical state, UFH can be prescribed in cases in which it is considered that there is an increased risk of bleeding and an invasive procedure is necessary. When using UFH, bear in mind the risk of contamination associated with the need to take serial blood samples. LMWH can be used at a dosage of 1 mg/kg 2x/day to protect the care team from the risk of contamination associated with using UFH. Patients in hospital in a serious or critical condition should have prothrombin activity time (PAT), APTT, DD, fibrinogen, and fibrin degradation products measured routinely, since elevation of these markers is associated with worse prognosis and a high prevalence of VTE and death.^3^^,^^6^^,^^11^^,^^13^^-^21

Indications for VDUS should be the same as those for patients without COVID-19 and this examination should only be performed if the result will be decisive for choice of therapeutic management. Elevated DD levels should not be the only factor in deciding to use VDUS.^11^^-^14 Preference should be given to use of portable and wireless ultrasound and the point-of-care technique to reduce the risk of contamination.^11^^-^14 Post-mortem studies have demonstrated a high prevalence of pulmonary microthrombosis in COVID-19-positive patients who die.^15^^,^^16^ Apparently, all types of heparin are able to reduce DD levels and mortality.^1^^,^^3^^,^^6^^,^^13^^-^21

Full-dose anticoagulation can be considered for patients with clinical suspicion of VTE and elevated DD (> 3000 mg/dL) and for those who exhibit accentuated increases in levels, as long as there is no absolute contraindication.^13^^,^^14^ Risks and benefits should be considered on a case-by-case basis. This conduct apparently offers benefits in terms of reduced length of stay in the ICU and reduced mortality. Published studies have not reported an increased risk of hemorrhage associated with this approach; however, it should be remembered that published studies are scarce and the quality of the scientific evidence available is low.^1^^,^^3^^,^^6^^,^^13^^-^21 Any significant clinical change in the progress of COVID-19 patients should be monitored and the hypothesis of VTE and TPE should be considered.^1^^,^^3^^,^^6^^,^^10^^,^^13^^-^18^,^^21^^-^24 The availability of hospital resources and the potential for contamination should be evaluated when choosing tests and treatment tactics. Empirical anticoagulation can be considered in the following situations, if supplementary test results are not available: increased DD (> 500 ng/mL or constant increase on 2 consecutive days); O_2_ saturation < 88% at admission and progressive demand for ventilatory support. In these situations, it is considered that the risk of TPE is high.^1^^,^^3^^,^^6^^,^^10^^,^^13^^-^16^,^^18^^,^^21^^-^24 In critical patients, if there are signs suggestive of massive or submassive TPE, sudden exacerbation of hemodynamic parameters or ventilation, a bedside echocardiogram should be assessed and, if signs of right ventricle overload are found, systemic fibrinolysis or pharmacomechanical thrombectomy should be considered, taking into account the risk of bleeding.^1^^,^^3^^,^^6^^,^^10^^,^^13^^-^21 For hospitalized patients, in the case of hemodynamic collapse, cardiac shock, or extracorporeal circulatory support (oxygenation by membrane), pharmacomechanical or surgical thrombectomy should be considered.^1^^,^^3^^,^^6^^,^^10^^,^^13^^-^21 If there is a clinical or radiological diagnosis of VTE while in hospital, full anticoagulation should be maintained for at least 3 months.^13^^,^^14^ At hospital discharge, for patients at high risk of VTE (age ≥75 years; > 60 years and DD > twice the reference value; 40-60 years and DD > twice the reference value and a history of VTE or cancer), with a Caprini score > 8 or an International Medical Prevention Registry on Venous Thromboembolism (IMPROVE-VTE)score > 4, it is recommended that pharmacological VTE prophylaxis be maintained for at least 6 weeks.^9^^,^^10^^,^^13^^,^^14^^,^^22^^,^^23^

## WHICH ANTICOAGULANTS SHOULD BE USED AFTER HOSPITAL DISCHARGE?

After hospital discharge, LMWH or DOAC can be used; DOAC may be preferred because it offers greater therapeutic convenience. The rivaroxaban dose to be given via the oral route (OR) should be 10 mg/day, OR, for 31 to 39 days (dose and indication approved by the Food and Drug Administration [FDA]).^23^^,^^24^

VTE occurs frequently in hospitalized patients, particularly among those in a serious condition being treated in an ICU. Studies indicate that SARS-CoV-2 is associated with an exacerbated inflammatory process, coagulopathy, and higher risk of cardiovascular mortality. The high risk of contagion and hemodynamic instability make it difficult to diagnosis and define the true prevalence of VTE in COVID-19. Therefore, definition of specific models for risk stratification, prophylaxis, and treatment of VTE during the pandemic is a real challenge. There is an urgent need for multicenter randomized clinical trials that can provide reliable data to guide conduct and protocols. However, we must not forget that COVID-19 is an emerging, severe, infectious pandemic for which there is not yet a specific treatment.^25^ Apparently, VTE is a common complication and is related to the most severe cases and to mortality. Use of LMWH in prophylactic and therapeutic doses has been demonstrating clinical benefits and low risk of hemorrhagic complications. It can therefore be concluded that there is an elevated risk of VTE during the COVID-19 pandemic and that prophylaxis and treatment with heparin in its several different presentations and dosages should be provided aggressively for patients who are not at high risk of hemorrhagic complications.
