# Supplementary material for: Venous thromboembolism in COVID-19 patients
Source: J Vasc Bras. 2020 Oct 16;19:e20200107. doi: 10.1590/1677-5449.200107 (PMC8217998; doi:10.1590/1677-5449.200107)
Supplement: Translation

# Translation: Tromboembolismo venoso em pacientes COVID-19

**Authors:** Fabio Henrique Rossi

**Affiliations:** 1 Instituto Dante Pazzanese de Cardiologia de São Paulo – IDPC-SP, São Paulo, SP, Brasil.

**Keywords:** COVID-19, SARS-CoV-2, trombose, anticoagulante, coagulação intravascular disseminada, profilaxia

## Abstract

A Covid-19 é uma doença respiratória potencialmente grave causada pelo RNA vírus SARS-CoV-2, que apresenta risco aumentado de tromboembolismo venoso (TEV). Sua fisiopatologia está relacionada a processo inflamatório exacerbado e a coagulopatia associada, verificada pelo aumento do D-dímero, do fibrinogênio e dos produtos da degradação da fibrina. Sua ocorrência deve ser monitorada, prevenida e tratada de acordo com as recomendações e diretrizes existentes. Devido a sua associação com as formas mais graves da doença e morte, alguns grupos vêm propondo uma conduta profilática e terapêutica mais agressiva. Entretanto, não existe uma definição quanto ao risco-benefício desse tipo de conduta, devendo ser avaliada individualmente e de forma multidisciplinar. Neste estudo, revisamos os principais estudos e evidências disponíveis até o momento sobre o diagnóstico, profilaxia e recomendações de tratamento do TEV em pacientes COVID-19.

## INTRODUÇÃO

A pandemia Covid-19, causada pelo coronavírus SARS-CoV-2, além de poder provocar lesão pulmonar alveolar e falência respiratória aguda, apresenta uma elevada prevalência de doenças cardiovasculares, sobretudo tromboembolismo venoso (TEV)^1^^-^3. Esse aumento do risco parece estar particularmente associado a reação inflamatória exacerbada e liberação exagerada de citocinas, sobretudo interleucina 6^4^^,^^5^. A coagulopatia é verificada pelo aumento dos índices de fibrinogênio, D-dímero (DD), fator VIII e prolongamento do tempo de protrombina (TP) e do tempo de tromboplastina parcial ativada (TTPa), fatores associados a má evolução clínica e óbito^6^^-^8.

Esses distúrbios ocorrem sobretudo em pacientes portadores de fatores de risco, como idade avançada, obesidade, hipertensão arterial sistêmica, *diabetes mellitus*, cardiopatias, pneumopatias, câncer, trombofilias, história prévia de TEV e outras comorbidades, mas também em crianças e indivíduos mais jovens, sugerindo haver um componente genético envolvido. Além disso, a imobilização, desidratação e necessidade de ventilação mecânica são fatores que podem contribuir para a alta prevalência de TEV na COVID-19. Apesar de essa prevalência não estar definida nas diversas fases da doença, tem-se observado que, nas formas mais graves e principalmente nos pacientes internados em unidade de terapia intensiva (UTI), existe elevado risco de tromboembolia pulmonar (TEP)^1^^-^3^,^^6^^-^8.

As recomendações aqui apresentadas são baseadas em consensos internacionais para a profilaxia e o tratamento de TEV em pacientes não COVID-19^9^^,^^10^, em editoriais, estudos retrospectivos de séries de casos e recomendações e opiniões de especialistas publicadas recentemente em regime de urgência, a maioria sem revisão por pares, sobre a prática clínica em pacientes COVID-19^11^^-^14. Dessa forma, devem ser aplicadas de forma individualizada e por equipe multidisciplinar experiente e treinada no manuseio dessas medicações e procedimentos.

A terapia anticoagulante está associada a benefícios substanciais (redução de risco de extensão de trombo e TEP fatal na doença aguda e recorrência de TEV). Nosso objetivo é apresentar, de forma objetiva e prática, recomendações baseadas nas evidências atuais sobre o diagnóstico, profilaxia e tratamento do TEV em pacientes COVID-19.

## DIAGNÓSTICO DO TEV EM PACIENTES COVID-19

Pacientes com suspeita clínica de COVID-19, mesmo tratados em regime domiciliar e especialmente aqueles que apresentem febre, anorexia e diarreia, devem ser orientados sobre a importância da hidratação, alimentação saudável e exercício dos membros. Na ocorrência de suspeita clínica de TEV (dor e edema de membros inferiores, dor torácica, piora súbita da dispneia etc.), o paciente deve ser orientado a procurar, via telemedicina, um angiologista ou cirurgião vascular e, caso necessário, realizar consulta presencial. Hoje, a maioria das clínicas é equipada com equipamentos de eco-Doppler vascular (EDV), que pode auxiliar no diagnóstico. Em pacientes tratados em regime domiciliar, não existe indicação para a solicitação de exames laboratoriais de rotina com o objetivo de identificar a presença de coagulopatia ou aumento de DD. Nos pacientes internados, a maioria dos autores recomenda que esses dados sejam checados periodicamente, apesar de não haver estudos que tenham verificado a custo-efetividade de tal prática^3^^,^^4^. Nesses pacientes, o diagnóstico do TEV por métodos de imagem complementares pode ser problemático durante o curso de uma pandemia devido à exaustão dos recursos hospitalares, além do risco de contaminação daqueles não COVID positivo, sobretudo daqueles internados em UTI, e da equipe envolvida no tratamento^13^^,^^14^.

Nos pacientes que apresentam DD elevado ou suspeita clínica elevada de TEP e quando houver dificuldade de acesso a tomografia, tem sido recomendada a realização de exames complementares à beira do leito. O ecocardiograma transtorácico é capaz de identificar sinais de sobrecarga de ventrículo direito, que podem sugerir a presença de TEP. O EDV, realizado pela técnica *point-of-care*, pode verificar a presença de trombose venosa profunda no eixo fêmoro-poplíteo^13^^,^^14^.

## PROFILAXIA EM PACIENTES NÃO INTERNADOS

Os pacientes que fazem uso de antitrombóticos devem ser orientados a continuar o uso desse medicamento, mas devem ser alertados sobre o risco de interação medicamentosa entre esses e medicamentos prescritos para a COVID-19, sobretudo os antivirais e anti-inflamatórios hormonais e não hormonais. De uma forma geral, não existe indicação para a farmacoprofilaxia do TEV; entretanto, a profilaxia com heparina de baixo peso molecular (HBPM) pode ser considerada, sobretudo nos portadores de risco aumentado para TEV (escore de Caprini > 8)^11^^-^14, desde que não haja risco aumentado para sangramento. A HBMP deve ser priorizada por apresentar meia vida mais curta e menor interação medicamentosa que os anticoagulantes orais diretos (DOACs). O uso de antagonistas da vitamina K (AVK) deve ser evitado pela dificuldade de controle dos níveis do índice internacional normalizado (INR), devendo-se administrar DOACs em seu lugar sempre que possível. Entretanto, pacientes portadores de válvulas mecânicas cardíacas, fibrilação atrial valvar síndrome do anticorpo antifosfolípide ou pacientes que estejam amamentando devem continuar usando AVK^13^^,^^14^. Deve ser considerada a existência de interação medicamentosa entre os diversos agentes anticoagulantes com os medicamentos de uso contínuo e os recomendados para o tratamento da COVID-19. A universidade de Liverpool disponibiliza em seu site uma extensa lista de possíveis interações entre os medicamentos frequentemente utilizados na COVID-19.

## PROFILAXIA E TRATAMENTO EM PACIENTES INTERNADOS

Em todos os pacientes internados com diagnóstico confirmado ou suspeita clínica de COVID-19, existe risco elevado de TEV. Todos devem receber farmacoprofilaxia, caso não haja contraindicação absoluta^9^^,^^10^^,^^13^^,^^14^. Nos pacientes que apresentam risco aumentado (escore de Caprini > 8), recomenda-se o uso de dose aumentada ou dobrada de profilaxia e, na ocasião da alta hospitalar, a extensão da farmacoprofilaxia parenteral ou oral por até 30 dias^13^^,^^14^. Entretanto, devemos enfatizar que essa prática não é fundamentada em estudos clínicos e deve ser aplicada de forma individualizada, considerando os riscos e benefícios em cada caso específico.

## DOSES RECOMENDADAS^13^^,^^14^

A dose regular de HBPM, subcutânea (SC), é de 30 mg 2x/dia ou 40 mg/dia. Para pacientes com obesidade [índice de massa corporal (IMC) > 30 kg/m^2^), escore de Caprini > 8 e DD elevado (200-3500 ng/mL), considerar 60 mg, SC, 2x/dia; para pacientes com insuficiência renal (*Clearence* de creatinina < 30 mL/min), considerar heparina não fracionada (HNF) 5000 UI, SC 3x/dia; para paciente com história de trombocitopenia induzida por heparina, considerar Fondaparinux, 2,5 a 5 mg, SC, 1x/dia. Para aqueles com contagem de plaquetas < 30.000 ou contraindicação absoluta para anticoagulação, usar dispositivos de compressão pneumática intermitente. Para pacientes em estado crítico, a HNF pode ser indicada nos casos em que se considera haver risco aumentado de sangramento e necessidade de procedimento invasivo. No uso da HNF, considerar o risco de contaminação associado à necessidade de coleta de amostra sanguínea seriada. A HBPM pode ser usada na dosagem de 1 mg/kg 2x/dia para proteger a equipe do risco de contaminação, associada ao uso de HNF. Pacientes internados em estado grave ou crítico devem ter os níveis de tempo de atividade da protrombina (TAP), TTPa, DD, fibrinogênio e produto de degradação de fibrina mensurados, rotineiramente, uma vez que a elevação desses marcadores está associada a pior prognóstico e a alta prevalência de TEV e óbito^3^^,^^6^^,^^11^^,^^13^^-^21.

As indicações para a realização de EDV devem ser as mesmas daquelas para pacientes não COVID-19, e esse exame só deve ser realizado se seu resultado for decisivo para a determinação da conduta terapêutica. O nível aumentado do DD não deve ser o único fator para a realização do EDV^11^^-^14. Deve ser dada preferência a equipamento de ultrassom portátil e sem fio e a técnica *point-of-care* para diminuir os riscos de contaminação^11^^-^14. Estudos necroscópicos vêm demonstrando alta prevalência de microtrombose pulmonar em pacientes COVID-19 positivo que evoluíram a óbito^15^^,^^16^. Aparentemente, todas as heparinas são capazes de diminuir os níveis de DD e a mortalidade^1^^,^^3^^,^^6^^,^^13^^-^21.

Pode-se considerar a anticoagulação em dose plena para os pacientes com suspeita clínica de TEV e dosagem elevada de DD (> 3000 mg/dL) e para aqueles que evoluam com aumento acentuado dos valores, desde que não haja contraindicação absoluta para tal^13^^,^^14^. Os riscos e benefícios devem ser considerados individualmente. Essa conduta aparentemente é benéfica ao reduzir o tempo de UTI e mortalidade. Os estudos publicados não verificaram aumento do risco de hemorragia com esse tipo de conduta; entretanto, deve-se levar em conta a escassez de estudos publicados e a baixa qualidade da evidência científica^1^^,^^3^^,^^6^^,^^13^^-^21. Qualquer alteração clínica significativa na evolução do paciente COVID-19 deve ser monitorada e considerada a hipótese de ocorrência de TEV e TEP^1^^,^^3^^,^^6^^,^^10^^,^^13^^-^21. Deve-se avaliar a disponibilidade de recursos hospitalares e o potencial de contaminação na escolha dos exames complementares e da tática terapêutica. A anticoagulação empírica pode ser considerada nas seguintes situações, caso não haja acesso a exames complementares: aumento de DD (> 500 ng/mL ou aumento constante em dois dias consecutivos); saturação de O2 < 88% na admissão e necessidade progressiva de suporte ventilatório. Nessas situações, considera-se que o risco de TEP é alto^1^^,^^3^^,^^6^^,^^10^^,^^13^^-^21. Nos pacientes críticos, se houver sinais sugestivos de TEP maciço ou submaciço, piora súbita dos parâmetros hemodinâmicos ou do padrão ventilatório, deve-se avaliar a indicação de ecocardiograma à beira do leito e, se forem identificados sinais de sobrecarga no ventrículo direito, deve-se considerar a fibrinólise sistêmica ou trombectomia farmacomecânica, considerando-se o risco de sangramento^1^^,^^3^^,^^6^^,^^10^^,^^13^^-^21. Nos pacientes internados, caso haja colapso hemodinâmico, choque cardíaco ou suporte circulatório extracorpóreo (oxigenação por membrana), deve ser considerada a trombectomia farmacomecânica ou cirúrgica^1^^,^^3^^,^^6^^,^^10^^,^^13^^-^21. Se, durante a internação, houver diagnóstico clínico ou radiológico de TEV, deve ser mantida anticoagulação plena por pelo menos 3 meses^13^^,^^14^. Na alta hospitalar, nos pacientes em que for identificado risco alto para TEV (idade ≥75 anos; > 60 anos e DD > duas vezes o valor de referência; 40-60 anos e DD > duas vezes o valor de referência e história de TEV ou câncer), escore de Caprini > 8 ou *International Medical Prevention Registry on Venous Thromboembolism* (IMPROVE-VTE) > 4, recomenda-se farmacoprofilaxia por pelo menos 6 semanas^9^^,^^10^^,^^13^^,^^14^^,^^22^^,^^23^.

## QUAL ANTICOAGULANTE USAR APÓS A ALTA HOSPITALAR?

Após a alta hospitalar, podem ser usados HBPM ou DOAC, e DOACs podem ser preferidos por apresentarem melhor comodidade terapêutica. A dose de rivaroxabana a ser utilizada via oral (VO) deve ser de 10 mg/dia, VO, por 31 a 39 dias [dose e indicação aprovada pela *Food and Drug Administration* (FDA)]^23^^,^^24^.

O TEV ocorre frequentemente em pacientes hospitalizados, sobretudo naqueles em estado grave e tratados em UTI. Estudos apontam que o SARS-CoV-2 é associado a processo inflamatório exacerbado, coagulopatia e maior risco de mortalidade cardiovascular. O alto risco de contágio e a instabilidade hemodinâmica dificultam o diagnóstico e a definição da real prevalência do TEV na COVID-19. Dessa forma, o estabelecimento de modelos específicos para a estratificação dos riscos, de profilaxia e de tratamento do TEV durante a pandemia representa um verdadeiro desafio. Existe uma necessidade urgente de estudos clínicos, multicêntricos e randomizados, que possam trazer dados confiáveis para nortear condutas e protocolos. Entretanto, devemos considerar que a COVID-19 é uma pandemia infecciosa grave, recente e para a qual não existe, até o momento, um tratamento específico^25^. Aparentemente, o TEV representa uma complicação frequente e relacionada a casos mais graves e óbito. O uso de HBPM em doses profiláticas e terapêuticas vem demonstrando benefício clínico e baixo risco de complicações hemorrágicas. Dessa forma, concluo que existe elevado risco de TEV na pandemia COVID-19 e que a profilaxia e o tratamento com o uso de heparina em suas diversas formas posológicas devem ser realizados de forma agressiva nos pacientes que não apresentem risco elevado de complicações hemorrágicas.
